# Co-Design Of Technology Driven Self-Management With Older Adults: An International Approach

**DOI:** 10.1093/geroni/igab046.2480

**Published:** 2021-12-17

**Authors:** Danielle Saney, Alessandro Pollini, Sandra Suijkerbuijk, Regina Krijger, Arlene Astell

**Affiliations:** 1 University of Toronto/Toronto Rehabilitation Institute, Toronto, Ontario, Canada; 2 BSD Design, Rome, Lazio, Italy; 3 Vilans, Utrecht, Utrecht, Netherlands; 4 Careyn, Utrecht, Utrecht, Netherlands; 5 University of Toronto, Toronto, Ontario, Canada

## Abstract

There has been an emergence in technology applications (apps) aimed at addressing needs amongst older adults and persons with cognitive impairment (PwCI). Despite the ubiquity of these apps, utilization is low, primarily due to a lack of involvement of PwCI and the perception that these apps have little motivational value. Engaging PwCI in creative processes such as co-design could lead to the creation of apps that better meets the needs of this population. The current study applies a user-centered, participatory approach to involve PwCI in the design of a new self-management and coaching app, RESILIEN-T. Co-design workshops were held with 12 PwCI across Italy, Netherlands and Canada; structured as four modules: (1) introduction and expectation setting, (2) user analysis, (3) storytelling, and (4) collaborative design. Based on interviews with PwCI, stories of individuals which reflect the target population were created (personas) and the solutions to the needs of these personas were discussed. Information about participant’s interests, computer proficiency and self-rated cognitive decline were collected. Participants were asked to try 10 existing apps and provided feedback on the design, usability and functionality. Lastly, participants were shown a prototype for RESILIEN-T and provided feedback based on the personas that they helped create. Co-design activities revealed that personalization is crucial for adherence. Aspects of physical and social activity, nutrition and cognition were most important to participants. Participants found many apps that are recommended for older adults do not appear age appropriate and seem condescending. These findings were common across PwCI from various nations.

